# Residual levels and dietary intake risk assessment of 11 pesticides in apricots from different ecological planting regions in China

**DOI:** 10.1038/s41598-022-23564-4

**Published:** 2022-11-05

**Authors:** Song Yang, Yujun Xing, Quanquan Liu, Hairong Wang, Aiguo Gu, Jinzheng Wang, Xiaomin Xue, Ru Chen

**Affiliations:** 1 Shandong Institute of Pomology, 66 Longtan Street, Taian, Shandong 271018 People’s Republic of China; 2 Jiangsu Product Quality Testing & Inspection Institute, 5 Guanghua Street, Nanjing, Jiangsu 210007 People’s Republic of China; 3 Jiangsu Key Laboratory for Food Quality and Safety/Institute of Food Safety and Nutrition, Jiangsu Academy of Agricultural Sciences, Nanjing, 210014 People’s Republic of China; 4 Dongying Natural Resources and Planning Bureau, 95 Fuqian Street, Dongying, Shandong 257000 People’s Republic of China

**Keywords:** Drug discovery, Ecology, Evolution, Environmental sciences, Risk factors

## Abstract

The frequent and massive use of pesticides has led to pesticide residues in apricot, threatening food safety and human health. A reliable and simple modified QuEChERS method with ultra-performance liquid chromatography-tandem mass spectrometry was developed for the simultaneous determination of 11 pesticides in apricot. Method validation indicated that satisfied linearity (R^2^ ≥ 0.9959), accuracy (recoveries of 72–119%), sensitivity (limits of detection, 0.03–0.30 μg/kg; limits of quantification, 0.13–1.00 μg/kg), and precision (relative standard deviations ≤ 11.9%), and matrix effects were 0.89–1.13. Apricot samples from different ecological regions in China were collected and tested using the proposed methods. Monitoring results were used to assess the dietary intake risk of Chinese populations of different ages and genders. Dietary risk assessment revealed that the risk quotients were 0.003–1.184% for different gender and age groups in China, indicating none unacceptable public health risk for general population. This work was thus significant in developing a simpler, more efficient and economical analysis method and food safety risks of the 11 pesticides on apricot and facilitated the establishment of maximum residue limits.

## Introduction

Apricots are native to China, and both the flesh and nucleoli are edible^1^. Fresh apricots taste sweet and sour, very palatable, and are widely favored by consumers in China and other countries. Because of apricot trees are often infected by pests such as apricot bees and diseases including apricot black spot, apricot shell scale insects, and so on^2,3^, since 2014, the apricot planting area in China has been growing slowly each year^4^. To increase the yield of apricots, fruit farmers often use excessive chemical pesticides for pest control, which not only seriously impacts the ecological environment but also leads to excessive pesticide residues in apricots, adversely affecting the health of consumers. Thus, various countries or organizations worldwide have established more strict maximum allowable residue limits (MRLs) for pesticides in apricots. Among these, the European Union (EU) and Japan have set the minimum limit requirements for 510^5^, and 312^6^ pesticide residues in apricots, respectively. However, at present, China has established limits for only 99 pesticide residues in apricots^7^, which is still significantly less.

Currently, there were relatively few studies on the methods of analysis of pesticide residues in apricots; only Li et al.^8^ have reported the determination of pesticide residues in Xiaobai apricots by ultra-performance liquid chromatography-tandem mass spectrometry (UPLC-MS/MS). Likewise, there is no report on the dietary risk assessment of pesticide residues in apricots. Therefore, it is necessary and imminent to establish an accurate, efficient, and sensitive analytical method that can simultaneously detect multiple pesticide residues in apricots and can be used to research dietary intake risk assessment. At present, there were many reports on the determination of pesticide residues in other fruits^9,10^, however, the analytical methods of pesticide residues in apricots were rarely reported. The pretreatment method is important for the determination of pesticide residues in fruits^11^. The usually used pretreatment methods for detection of pesticide residues in apricots include solid-phase extraction^12^, gel permeation chromatography (GPC)^13^, matrix dispersion extraction^14^, solid-phase microextraction^15^, supercritical fluid extraction (SFE)^16^, and the quick, easy, cheap, effective, rugged, and safe (QuEChERS) method^17^. Among them, GPC and SFE are relatively expensive, thus limiting their use in sample pretreatment^18^. QuEChERS technology combines sample extraction and matrix purification with the advantages of simple and rapid operation, high throughput, and low cost, and has become a commonly used pre-processing technology used in domestic and foreign laboratories^19,20^. Purification agents in the QuEChERS method include *N*-primary secondary amine (PSA), octadecyl alkyl (C_18_), and graphitized carbon black (GCB)^21,22^. Among these, PSA and C_18_ can effectively remove polar compounds such as fatty acids, organic acids, sugars, and polar pigments, as well as weakly polar compounds such as oils, proteins, fat-soluble vitamins, and fats. Yet, PSA and C_18_ have the disadvantages of poor adsorption stability due to the influence of pH, temperature, and other factors^23^, large usage, and high cost^24^. GCB has a planar hexagonal structure similar to graphite, which is strongly adsorbed on pesticide compounds with planar structure, thus affecting the recovery rate of pesticides with planar structure^25^. In recent years, nanomaterials have been gradually applied as adsorption materials in the pretreatment methods, because of their larger specific surface area, adsorption stability, and adsorption capacity^26^. For instance, nanometer zirconia (nano-ZrO_2_) and multi-walled carbon nanotubes (MWCNTs) have been used in the detection of veterinary drug residues^27^.

Pesticide residues in food products are a global concern because their negative impacts on human health depend on the means and amount of exposure^28,29^. Thus, it is critical to monitor pesticide levels in fruits and vegetables using all analytical techniques available^30,31^. A dietary risk assessment of pesticide residues in apricots will provide a scientific basis for adequate supervision, safe production, consumption guidance, and issuance and revision of MRLs^32^. This process will subsequently aid in eliminating adverse effects on the health of the consumers and foreign trade of apricot-based products. However, until now, no study has reported the risk associated with the dietary intake of pesticide residues in apricots.

In this study was designed to determine the residues of 11 pesticides in apricot to address food safety concerns. We developed and validated a sensitive and straightforward method to simultaneously detect and quantify 11 pesticides in apricot using a combination of nano-ZrO_2_, PSA, C_18_, and MWCNTs as purification agents. Subsequently, the optimized analytical method was used to monitor the residue levels of 11 frequently used pesticides on apricot from different ecological planting regions in China. Finally, we successfully assessed the dietary risk of pesticide residues in apricot according to the terminal residues and toxicological data.

## Materials and methods

### Reagents and materials

Reference standards (with purity over 98%) of pesticides including abamectin (B1a), imidacloprid, chlorpyrifos, *β*-cypermethrin, phoxim, procymidone, acetamiprid, deltamethrin, fenpropathrin, bifenthrin, and diflubenzuron were purchased from the Agriculture Environmental Protection Institute in Tianjin.

Deionized water was obtained from the Milli-Q water purification system (Millipore, France). Methanol (MeOH) and acetonitrile (ACN) were of LC–MS grade and purchased from Merck (Darmstadt, Germany). Analytical grade sodium chloride (NaCl) and anhydrous magnesium sulfate (MgSO_4_) were purchased from Sinopharm (Shanghai, China). The purification agents, MWCNTs, PSA, C_18_, and nano-ZrO_2_ were purchased from Angela Technologies Co., Ltd. (Tianjin, China).

### Sample collection

Apricot samples were collected from different ecological planting regions (Shandong, Xinjiang, Hebei, Gansu, Shanxi, Henan, and Beijing) in China, from June to July 2020. The sampling sites in seven provinces or cities in China are shown in Fig. S1 (Supporting Information). In the orchard, 15 collection points were selected by S-shape or X-shape for sampling. In addition, samples were not collected at distances less than 1 m from the orchard boundary. The amount of collection samples should not be less than 3 kg, and it must be transported back to the laboratory within 8 h. If the laboratory cannot be reached within 8 h, it must be transported with the freezer or a car refrigerator. A total of 30 samples (3 kg each) were randomly collected from the harvested apricot of each prefecture-level province or city^33^. Samples were serially numbered and stored at − 20 °C.

### Sample preparation

Samples were prepared following a QuEChERS-based method and each sample was ground in a blender. Approximately 5 g each of the homogenized samples were taken in a 50 mL centrifuge tube, mixed with 10 mL of ACN, and shaken for 5 min. Subsequently, NaCl (1 g) and MgSO_4_ (4 g) were added to the tube and shaken vigorously for 3 min, followed by centrifugation at 5000 rpm for 3 min. Then, 1 mL of the extracted solution was transferred to a 2 mL centrifuge tube containing different sorbent mixtures (10 mg PSA/30 mg nano-ZrO_2_/5 mg MWCNTs and 100 mg MgSO_4_). After shaking (5 min on a mechanical shaker) and centrifugation (12,000 rpm for 2 min), the supernatant was filtered through a 0.22 μm membrane filter for UPLC-MS/MS analysis. The extraction and purification conditions to analyze the 11 pesticides in the apricot matrix were optimized using different purification combinations, as mentioned in Table S1 (Supporting Information).

### Standard solutions

The stock solution (1000 mg/L) of each pesticide was dissolved in high-performance liquid chromatography-grade ACN. A mixed standard solution (100 mg/L) was prepared by diluting the standard stock solutions. It was serially diluted to obtain a series of standard solutions at concentrations of 0.001, 0.005, 0.01, 0.05, 0.1 and 0.5 mg/L for standard curve and matrix effect evaluation experiments. All solutions were stored at 4 °C in the dark.

### Ultra-performance liquid chromatography-tandem mass spectrometry

Ultra-performance liquid chromatography-tandem mass spectrometry was performed on a Waters Acquity UPLC system (Waters, Milford, MA, USA) coupled to a Xevo TQ-S cronos triple quadrupole mass spectrometer (Waters, Milford, MA, USA) equipped with an electrospray ion source (ESI). The heating gas (air), nebulizing gas, and drying gas (N_2_) were supplied by an N_2_ gas generator (ATN-1050; Shimadzu, Kyoto, Japan). UPLC–MS/MS instrument was controlled by the software workstation of MassLynx 4.1 (Waters, Milford, MA, USA).

### Chromatography

An ACQUITY UPLC HSS C_18_ chromatography column (50 mm × 2.1 mm, particle size = 1.7 µm; Waters, Milford, MA, USA) was used to separate the pesticides at 35 ℃. Solvent A (aqueous solution containing 0.1% formic acid and 5 mmol/L ammonium acetate) and B (ACN) were employed for gradient elution with the following gradient program: 0–4 min, 10–95% B; 4–6 min, 95% B; 6–6.1 min, 95–10% B; 6.1–8 min, 10% B. The flow rate was 0.35 mL/min, and the injection volume was 2 μL.

### Mass spectrometry

The MS/MS parameters were as follows: the target analytes were detected in positive mode and the capillary voltage was 0.5 kV; the temperature of the heated block was 450 ℃ with a flow rate of 800 L/h; cone gas flow rate, 30 L/h; ionization source temperature, 150 °C. The data acquisition was conducted in the multiple reaction monitoring (MRM) mode. These parameters were optimized by injecting individual standard solutions (500 ng/mL) of 11 pesticides. Analyte identification was based on the relative retention time and the relative percent ion ratio of the qualitative ion/quantitative ion. The parameters that affect the quantification and confirmation of analytes, including precursor-product ions, collision energies, and deviations, were optimized and listed in Table S2 (Supporting Information).

### Validation of the detection and quantification methods

These were performed according to the guidance document on method validation and quality control procedures for the analysis of pesticide residues in food and feed^34^, including specificity investigation, calibration curve, recovery (precision and accuracy), the limit of detection (LOD), the limit of quantification (LOQ), stability, and matrix effects (MEs).

### Linearity range and matrix effects

Linearity was assessed in both standard working solutions, as well as in the spiked standard samples, prepared by spiking standards into the extracted sample. Linearity of the method for 11 pesticides was verified using standards at six different concentrations (0.001, 0.005, 0.01, 0.05, 0.1, and 0.5 mg/L). The relative peak areas of analytes were plotted versus known concentrations, and the regression equations and their coefficients of determination (R^2^) were calculated. The MEs were investigated by analyzing the slopes of standard curves drawn between solvent-only and matrix-matched standard samples. The ME for each pesticide was calculated as follows:

1 $$ {\text{ME }}\left( \% \right) \, = {\text{ k}}_{{2}} /{\text{k}}_{{1}} \times { 1}00 $$

where k_1_ is the slope ratio of calibration curves with standards prepared with solvent and k_2_ is the slope ratio of matrix-matched calibration curves^35^.

### Recovery and precision

The accuracy and precision of the method were assessed using blank apricot samples spiked with 0.002, 0.02, 0.1, and 1 mg/kg concentrations of each pesticide, with six replicates each.

### Limit of detection and limit of quantification

The LOQ is the lowest validated level with sufficient recovery and precision while LOD is the lowest calibration level according to SANTE/12,682/2020^34^.

### Sample stability

To understand the stability of samples with 11 pesticides in apricot and ensure data reliability, two storage methods for blank apricot matrix in a range of concentrations (10, 100 µg/kg) of 11 pesticides were assessed. Stability was assessed by storing the samples at room temperature for 8 h (short-term) and − 20 °C for 30 d (long-term). The freeze–thaw stability was investigated in three cycles from − 20 °C to room temperature on three consecutive days. The stability of all samples was determined according to the above-mentioned procedure and analyzed by UPLC-MS/MS. The recoveries and RSD (%) of the tested samples were then calculated.

### Dietary risk assessment

The national estimated daily intake (NEDI; mg) and the risk quotient (RQ; %) of pesticide residues in apricots among the Chinese population were calculated as follows:

2 $$ {\text{NEDI }} = {\text{ STMR}}_{{\text{i}}} \times {\text{ F}}_{{\text{i}}} $$

3 $$ {\text{RQ }}\left( \% \right) \, = {\text{ NEDI}}/\left( {{\text{ADI }} \times {\text{ bw}}} \right) \, \times { 1}00 $$

where STMR_i_ (supervised trials *median* residue level; mg/kg) represents the median concentration of pesticide residues in apricot from China; F_i_ (kg) is the average daily intake of food in China; The NEDI represents the average daily dietary intake, obtained by multiplying STMRi and F_i_; ADI is the acceptable daily intake (mg/kg bw) of 11 pesticide residues; and bw is the average body weight of different age groups of the Chinese population. NEDI and ADI values were determined following good agricultural practice^36^. The RQ of each pesticide was determined using Eq. (2); RQ > 100% indicated unacceptable risk, and RQ ≤ 100% indicated acceptable risk^37^.

### Ethical approval

This article does not contain any studies with human participants or animals.

#### Statement of all methods:

We declared that all methods were carried out in accordance with relevant guidelines and regulations.

#### Statement of all experimental protocols:

We declared that all experimental protocols were approved by related institutional and/or licensing committee.

## Results and discussion

### Chromatographic separation and mass spectrometric optimization

To obtain the best monitoring conditions for each compound, a 0.5 mg/L mixed standard solution of 11 pesticides was mixed with the mobile phase through a syringe pump and then injected into the mass spectrometer for tuning. The precursor ion of the compound to be tested was determined by the primary mass spectrometry scan under ESI^+^ and ESI^-^ modes, and then the product ion was scanned by the secondary mass spectrometry. Two groups of ion pairs with the best sensitivity were selected for detection; one group was used for quantification, and another, for qualitative analysis. The optimization results showed high sensitivity of all the 11 pesticides under the ESI^+^ mode. Among them, abamectin (B1a), *β*-cypermethrin, deltamethrin, fenpropathrin, and bifenthrin were [M + NH_4_]^+^, and other compounds were [M + H]^+^. MS parameters of 11 pesticides are mentioned in Table S2.

**Figure 1:**
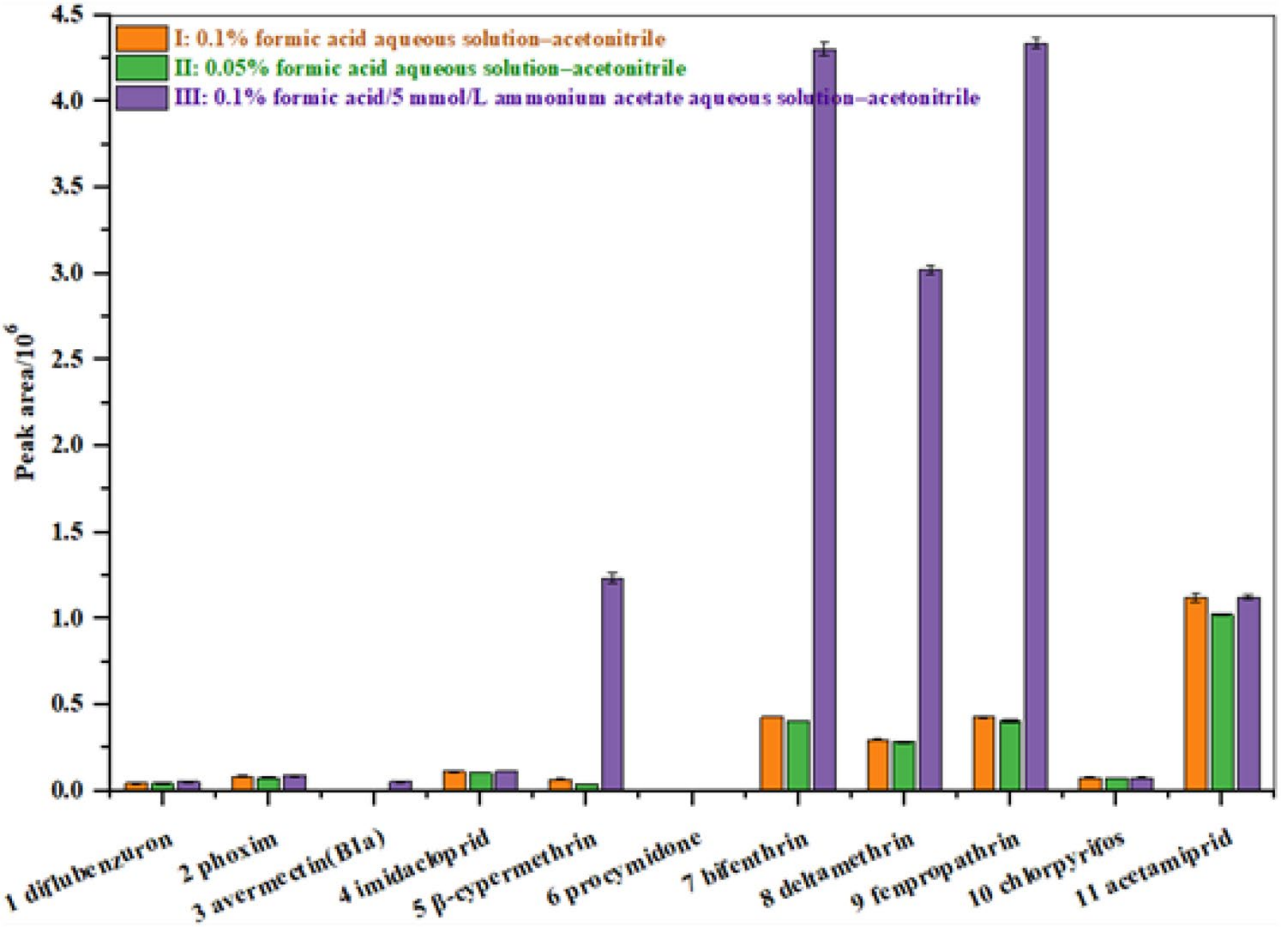
When using HSS C_18_, the peak areas of 11 pesticides in three different mobile phases.

Formic acid and ammonium acetate are commonly used reagents to enhance the ionization of target compounds [M + H]^+^ and [M + NH_4_]^+^ under the ESI^+^ mode, and they can effectively improve the peak pattern, making the peak sharper and more symmetrical; therefore, they need to be added during gradient elution^38^. To improve work efficiency, it is necessary to separate and complete the monitoring of 11 pesticides in the shortest possible time; therefore, we selected two different types of chromatographic columns (ACQUITY UPLC HSS C_18_ and ACQUITY UPLC HSS T3) and three different mobile phases (Ι: 0.1% formic acid aqueous solution—ACN, II: 0.05% formic acid aqueous solution—ACN, and III: 0.1% formic acid/5 mmol/L ammonium acetate aqueous solution—ACN) for optimization experiments. We observed that when using the HSS T3 chromatographic column, *β*-cypermethrin, deltamethrin, fenpropathrin, and bifenthrin did not show a good retention effect under the three mobile phase systems, and there was substantial tailing of the chromatographic peak. The shape of the chromatographic peak and sensitivity of the target compound were used as evaluation indicators. Compared with Ι and II, mobile phase III produced better sensitivity for all target compounds (Fig. 1), with sharper and more symmetrical peaks of *β*-cypermethrin, deltamethrin, fenpropathrin, and bifenthrin. This may be because the addition of 5 mmol/L ammonium acetate improved the retention performance of the HSS C_18_ chromatography columns without affecting the ionization efficiency of all target compounds. In summary, we selected the HSS C_18_ column for chromatographic separation and used 0.1% formic acid/5 mmol/L ammonium acetate aqueous solution—ACN as the mobile phase to further optimize the gradient elution procedure and effectively separate and detect all the target compounds within 8 min.

### Optimization of purification materials

The flesh of apricot contains sugar, protein, calcium, phosphorus, carotene, thiamine, riboflavin, niacin, and vitamin C. Due to these diverse impurities, the analysis of the sample matrix becomes highly complex. Therefore, these impurities need to be removed from the matrix samples before analysis. Currently, PSA, C_18_, and MWCNTs are widely used to adsorb to the fruit substrate^39^. PSA has a strong adsorption capacity for metal ions, fatty acids, sugars, and fat-soluble pigments, C_18_ has a strong adsorption capacity for non-polar impurities (such as fat, sterol, and volatile oil), while MWCNTs have a strong adsorption capacity for pigments, which can effectively remove chlorophyll, lutein, and carotene. However, C_18_ and MWCNTs can also simultaneously adsorb pesticides, resulting in poor recovery. Nano-ZrO_2_ has a large specific surface area and good adsorption stability and has recently been used to purify substrates. It can selectively remove fats and pigments from samples compared to conventional C_18_ fillers.

**Figure 2:**
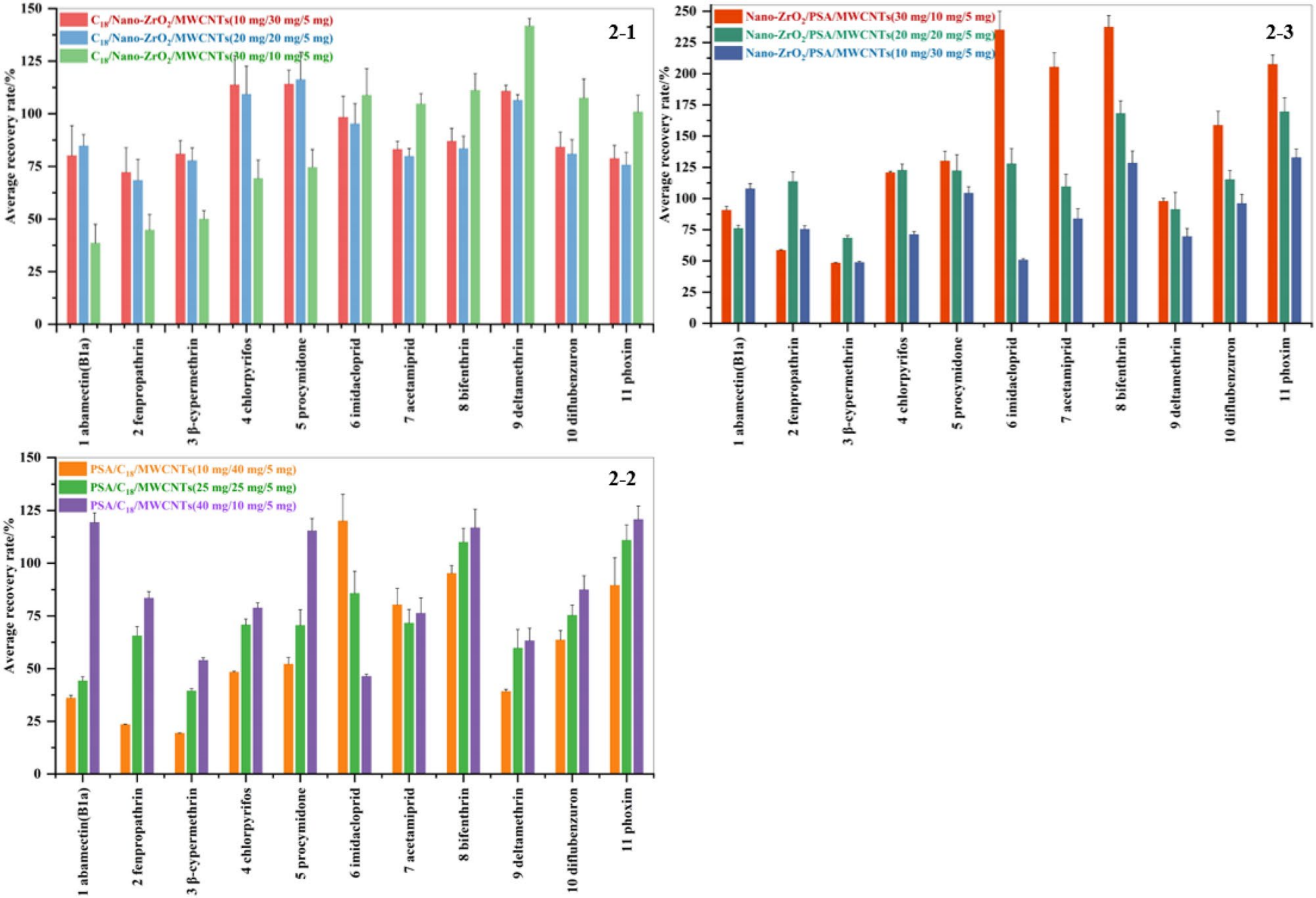
The recoveries of 11 pesticides in apricot matrix under different scavenger combinations (2–1 C_18_/nano-ZrO_2_/MWCNTs, 2–2 PSA/C_18_/MWCNTs, 2–3 nano-ZrO_2_/PSA/MWCNTs; 0.01 mg/kg, n = 3).

In the current study, different purification materials were combined for the analysis of 11 pesticide residues and to propose the best purification strategy in the pretreatment of apricot samples. As displayed in Fig. 2, the average recovery of 11 pesticides in the apricot was higher using the C_18_/nano-ZrO_2_/MWCNTs than other combinations. Nano-ZrO_2_ showed better adsorption than PSA in purifying fatty acids, organic acids, polar pigments, and sugars in apricot, owing to its larger specific surface area, better adsorption capacity, and stability. To conclude, the combination of 10 mg C_18_, 30 mg nano-ZrO_2_, and 5 mg MWCNTs demonstrated the best recovery for 11 pesticides, with recovery in the range of 72% to 114%, at a pesticide spiking level of 0.01 mg/kg. In summary, we finally determined that among the tested combinations, C_18_/nano-ZrO_2_/MWCNTs (10 mg/ 30 mg/5 mg) is the best purification combination for the pre-treatment of apricot samples.

### Linearity, matrix effects, limit of detection and limit of quantification

The standard curve obtained from the standard working solutions of 11 pesticides and the calibration curve from blank apricot matrix spiked with 11 pesticides showed good linearity (0.001, 0.005, 0.01, 0.05, 0.1, and 0.5 mg/L), with R^2^ ≥ 0.9959 for all tested samples (Table 1).

**Table 1:** The standard curves, R^2^ and MEs of 11 pesticides in apricot.

Pesticides	Matrixs	Linear equation	R^2^	MEs
1 Abamectin(B1a)	Acetonitrile (k_1_)	Y = 395 X − 1624	0.9990	1.12
	Apricot (k_2_)	Y = 444 X − 985	0.9991	
2 Phoxim	Acetonitrile (k_1_)	Y = 3698 X + 44,612	0.9965	0.89
	Apricot (k_2_)	Y = 3278 X + 17,136	0.9986	
3 Bifenthrin	Acetonitrile (k_1_)	Y = 6789 X + 25,090	0.9990	1.04
	Apricot (k_2_)	Y = 7073 X + 39,188	0.9973	
4 Chlorpyrifos	Acetonitrile (k_1_)	Y = 1057 X + 2112	0.9997	0.94
	Apricot (k_2_)	Y = 994 X + 2962	0.9995	
5 *β*-Cypermethrin	Acetonitrile (k_1_)	Y = 4892 X − 384	0.9998	0.99
	Apricot (k_2_)	Y = 4848 X + 4522	0.9996	
6 Imidacloprid	Acetonitrile (k_1_)	Y = 283 X − 524	0.9991	1.12
	Apricot (k_2_)	Y = 315 X − 517	0.9995	
7 Diflubenzuron	Acetonitrile (k_1_)	Y = 869 X + 369	0.9977	1.08
	Apricot (k_2_)	Y = 941 X − 7351	0.9959	
8 Acetamiprid	Acetonitrile (k_1_)	Y = 9709 X + 13,129	0.9965	1.04
	Apricot (k_2_)	Y = 10,121 X + 36,497	0.9982	
9 Fenpropathrin	Acetonitrile (k_1_)	Y = 7214 X + 3859	0.9997	1.02
	Apricot (k_2_)	Y = 7335 X + 1239	0.9991	
10 Deltamethrin	Acetonitrile (k_1_)	Y = 4455 X − 27,066	0.9991	1.02
	Apricot (k_2_)	Y = 4535 X − 26,815	0.9989	
11 Procymidone	Acetonitrile (k_1_)	Y = 24 X − 152	0.9969	1.13
	Apricot (k_2_)	Y = 27 X − 173	0.9970	

To evaluate MEs, the slopes of matching 11 pesticide standards with solvent and apricot matrix were calculated at the same concentration. According to the derived slope of the matrix-matched calibration curve, MEs of 11 pesticides in apricot were between 89 and 113% (Table 1), well within the range of 80% to 120%, indicating that the MEs could be ignored. It also suggests that the current pre-treatment method has a good purification effect and eliminates the matrix effect very well, laying a robust foundation for the subsequent step of quantitative analysis of samples. We next used the standard solution curve to quantify the 11 pesticide residues in apricot.

The LOD refers to the minimum concentration or minimum amount of a component to be tested that can be detected from a test sample under a given confidence level by an analytical method. Its physical meaning is the amount of the measured component when the signal is 3 times the standard deviation (S = 3σ) of the reagent blank signal (background signal). Sometimes it also refers to the amount of the measured component corresponding to when the signal is three times the background signal generated by the reagent blank (S = 3 N). The LOQ refers to the minimum amount of the analyte in the sample that can be quantitatively determined, and the determination result should have a certain accuracy^40^. The LOQ reflects whether the analytical method has the sensitive quantitative detection ability. The LOQ is the lowest validated level with sufficient recovery and precision, which was estimated to be 0.001 mg/L, while the LOD is the lowest calibration level, which was 2 µg/kg, according to SANTE/12,682/2020.

### Accuracy and precision

**Figure 3:**
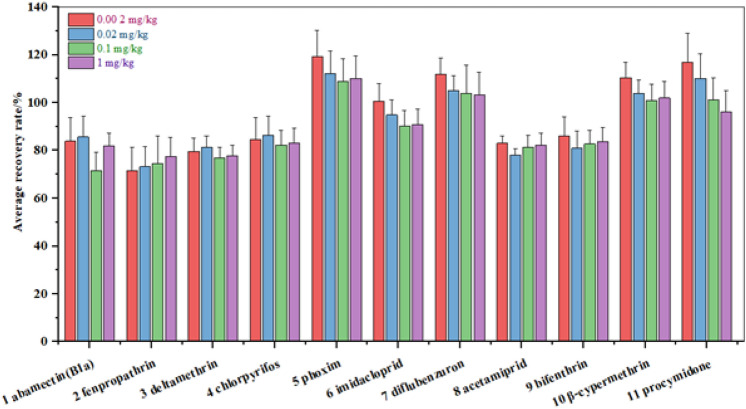
The recoveries and precisions of 11 pesticides were spiked at four levels in the apricots (n = 6).

In the matrix, 11 pesticides were spiked at four levels (0.002, 0.02, 0.1, and 1 mg/kg), and for each spiked sample, there were six replicates. The recoveries of 11 pesticides in apricot at all levels ranged between 72 and 119%. The inter- and intra-level relative standard deviations (RSDs, %) of 11 pesticides in apricot were < 11.9% (Fig. 3), suggesting that the method was reliable within reproducibility in the laboratory according to NY/T 788—2018^33^ and SANTE/12,682/2020^34^.

### Sample stability

The stability of samples during pre-treatment and UPLC-MS/MS was investigated by spiking the tested apricot samples with 11 pesticides at two concentrations. Under two storage conditions (room temperature for 8 h and at − 20 °C for 30 d), at the end of the test period, the recoveries of 11 pesticides at the two levels were 82%—117% (short-term) and 73%–103% (long-term), respectively (Table 2). Thus, the 11 pesticides showed good stability in the apricot matrix, making them suitable for subsequent analyses.

**Table 2:** The stability test results of 11 pesticides in apricot under two storage conditions (n = 3).

Pesticides	6 h (short-term, n = 3)				30 d (long-term, n = 3)			
	0.01 mg/kg		0.1 mg/kg		0.01 mg/kg		0.1 mg/kg	
	Recoveries/%	RSDs/%	Recoveries/%	RSDs/%	Recoveries/%	RSDs/%	Recoveries/%	RSDs/%
1 Abamectin(B1a)	107	8.5	111	12.7	103	7.7	102	11.9
2 Fenpropathrin	92	7.7	91	8.8	81	7.6	79	8.8
3 Deltamethrin	94	5.2	96	7.8	83	5.1	84	7.8
4 Chlorpyrifos	86	6.6	87	9.9	79	6.3	78	9.6
5 Phoxim	82	5.7	83	11.2	73	5.7	80	10.0
6 Imidacloprid	103	9.1	106	10.2	101	8.1	99	9.4
7 Diflubenzuron	84	6.9	94	5.2	81	6.3	87	4.9
8 Acetamiprid	91	4.1	100	5.8	80	4.0	88	5.8
9 Bifenthrin	117	8.3	96	7.1	103	8.3	84	7.1
10 *β*-Cypermethrin	110	6.7	111	9.3	97	6.6	97	9.3
11 Procymidone	93	6.5	106	9.2	83	6.4	97	8.7

### Terminal residues of 11 pesticides in apricots

The terminal residues of 11 pesticides in apricot samples collected from seven provinces or cities of China are listed in Table 3. Using the proposed method, four of the 11 pesticides, including acetamiprid, bifenthrin, fenpropathrin, and imidacloprid, were detected in apricot samples. The pesticide residues were detected in the samples from all sampling sites (Shandong, Xinjiang, Hebei, Gansu, Shanxi, Henan, and Beijing), with levels lower than the MRLs specified in China^7^. The research of Li et al.^8^ showed that imidacloprid was detected in Xinjiang apricots and below the MRLs^7^, which was consistent with our study. Therefore, imidacloprid is the high-frequency detection pesticide in apricot, which should be paid enough attention. Furthermore, only 41 out of the total 210 samples contained pesticide residues, with a detection rate of 19.5%. Therefore, to ensure food safety, the local government should strengthen monitoring and guidance of these detected pesticides specifically in those provinces or cities.

**Table 3:** The terminal residues of 11 pesticides in apricot samples collected from different ecological planting regions in China.

NO	Pesticides	Results of terminal residue (μg/kg, n = 3)	STMRs	HRs	MRLs (μg/kg)	
					China	EU
1	Abamectin(B1a)	ND	–	–	0.5	0.02
2	Fenpropathrin	11.5, 12.0, 17.6, 49.1, 57.1, 254.1	33.4	254.1	–	0.01*
3	Imidacloprid	6.3, 11.0, 11.1, 14.8, 16.4	11.1	16.4	0.5	0.5
4	Diflubenzuron	ND	–		–	0.01*
5	Acetamiprid	9.1, 13.1, 13.2, 13.2, 36.7, 50.4, 50.9, 55.89, 59.8, 64.4, 66.6, 66.9, 147.2, 151.1	53.4	151.1	2.0	0.8
6	*β*-cypermethrin	ND	–	–	0.5	2.0
7	Bifenthrin	14.5, 14.7, 26.9, 28.1, 31.8, 34.2, 86.9, 95.2	29.9	95.2	–	0.01*
8	deltamethrin	ND	–	–	0.05	0.15
9	chlorpyrifos	ND	–	–	3.0	0.01*
10	phoxim	ND	–	–	0.5	0.01*
11	procymidone	ND	–	–	–	0.01*

“STMR” is the supervised trials median residue. “HR” is the highest residue. “-” is not detected. “–” is the limit standard has not been established. “*” is the temporary maximum residue limits.

### Dietary risk assessment

To assess the possible exposure routes and levels of the pesticides, dietary exposure was conducted to clarify the actual/expected exposure and potential harm to sensitive groups^41^. For the dietary risk assessment, six groups (male and female aged 2–4 years, 18–30 years, and 60–70 years) were selected considering significant differences in the ratio of food intake to body weight among people of different genders and ages^42^. Considering the dietary needs of people and following the principle of maximizing risk, the intake of apricot was calculated based on the fruit intake by Chinese people (six groups). The average daily intake of apricots in the different groups is mentioned in Table 4. The NEDI and RQ were calculated according to the dietary intake and weight survey data combined with the pesticide residues (four pesticides in total) detected in apricots in our study. The estimated NEDI values of the four pesticides detected in apricots were in the range of 1.0 × 10^−4^ mg/d^.^bw to 25.2 × 10^−4^ mg/d^.^bw and the RQs of the four pesticides in apricot were 0.003–1.184% for Chinese people (Table 5). The sums of the RQs% of the four pesticides in apricot for Chinese people of different age groups and genders were 1.469% (2–4 yrs, male), 1.626% (2–4 yrs, female), 0.127% (18–30 yrs, male), 0.168% (18–30 yrs, female), 0.128% (60–70 yrs, male), and 0.162% (60–70 yrs, female). Thus, the RQ values were less than 100% and indicated an acceptable level of the four pesticides detected in apricots. Concurrently, in terms of gender, we found a higher risk of dietary exposure in women than in men; with increase in age, dietary exposure was observed to gradually decrease, while children (2–4 years old) had the highest dietary exposure. Meanwhile, the evaluation results showed that value of RQ of bifenthrin was highest, followed by acetamiprid, fenpropathrin and imidacloprid. Therefore, regulatory departments of government should strengthen the monitoring, supervision and regulation of bifenthrin to prevent the occurrence of events harmful to dietary health. However, the dietary risk assessment results of apricot samples collected from seven provinces or cities of China in this study were obtained from the total RQ values of detected pesticides. Obviously, this assessment method is still insufficient. In the future, we will study and develop a scientific model of dietary risk assessment of multiple pesticides that includes multiple factors.

**Table 4:** The F_i_ and body weight of apricot for different age groups in China.

Age	Gender	Bw/(kg)	F_i_/(kg/d)
2 ~ 4	Male	14.1	0.0437
	Female	13.4	0.0444
18 ~ 30	Male	60.5	0.0418
	Female	52.6	0.0529
60 ~ 70	Male	61.3	0.0338
	Female	54.3	0.0348

**Table 5:** The NEDIs and RQs% of 11 pesticides in apricot of different age groups in China.

Pesticides	ADIs (mg/kg)	Ages 2–4				Ages 18–30				Ages 60–70			
		Male		Female		Male		Female		Male		Female	
		NEDI (mg/d·bw) *10^4^	RQ%	NEDI (mg/d·bw) *10^4^	RQ%	NEDI (mg/d·bw) *10^4^	RQ%	NEDI (mg/d·bw) *10^4^	RQ%	NEDI (mg/d·bw) *10^4^	RQ%	NEDI (mg/d·bw) *10^4^	RQ%
1 Fenpropathrin	0.03	5.3	0.125	5.6	0.139	2.0	0.011	2.3	0.014	2.0	0.011	2.3	0.014
2 Bifenthrin	0.01	15.1	1.070	15.9	1.184	5.6	0.092	6.4	0.122	5.7	0.093	6.4	0.118
3 Imidacloprid	0.06	2.6	0.031	2.7	0.034	1.0	0.003	1.1	0.004	1.0	0.003	1.1	0.003
4 Acetamiprid	0.07	23.9	0.243	25.2	0.269	8.9	0.021	10.2	0.028	9.0	0.021	10.2	0.027
Total	–	–	1.469	–	1.626	–	0.127	–	0.168	–	0.128	–	0.162

## Conclusions

In summary, the proposed method was reliable for determining 11 pesticides in apricot. Notably, the extraction and cleanup procedures were optimized to obtain a quick and high recovery of 11 pesticides. Validation results of the proposed method provided good linearity (R^2^ ≥ 0.9959), sensitivity (limits of detection, 0.03–0.30 μg/kg; limits of quantification, 0.13–1.00 μg/kg), accuracy (recoveries of 72%—119%; relative standard deviations ≤ 11.9%), acceptable stability, and no MEs (0.89—1.13), making it a robust and well-suited method for detecting 11 pesticides at trace levels in apricot. The 11 pesticides showed good stability in the apricot matrix, making them suitable for subsequent analyses. Using the proposed method, four of the 11 pesticides, including acetamiprid, bifenthrin, fenpropathrin, and imidacloprid, were detected in apricot samples. The dietary exposure and risk assessment of pesticide residues among the representative Chinese population indicated a permissible chronic risk exposure (< 1.63%). Thus, the dietary intake of 11 pesticide residues does not pose a potential health risk to apricot consumers in China. In this study the dietary risk assessment results of apricot samples collected from seven provinces or cities of China in this study were obtained from the total RQ values of detected pesticides. However, factors such as pesticide toxicity, residue limits, and dietary structure of residents in different economic regions were not involved, so the final evaluation results may be different from the actual situation. In the future, we will study and develop a scientific model of dietary risk assessment of multiple pesticides that includes multiple factors.

## Supplementary Information

Supplementary Information.

## Data Availability

The data that support the findings of this study are available from the responding author upon reasonable request.
