# Supplementary material for: Residual levels and dietary intake risk assessment of 11 pesticides in apricots from different ecological planting regions in China
Source: Sci Rep. 2022 Nov 5;12:18818. doi: 10.1038/s41598-022-23564-4 (PMC9637227; doi:10.1038/s41598-022-23564-4)
Supplement: Supplementary file 1 — Supplementary Information. [file 41598_2022_23564_MOESM1_ESM.docx]

**Supporting Information**

**Residual levels and dietary intake risk assessment of 11 pesticides in apricots** **from different ecological planting regions in China**

Song Yang^2^, Yujun Xing^3^, Quanquan Liu^4^, Hairong Wang^1^, Aiguo Gu^2^, Jinzheng Wang^1^, Xiaomin Xue^1^, Ru Chen^1,^*

^1^ Shandong Institute of Pomology, 66 Longtan Street, Taian, Shandong Province 271018, P.R. China.

^2^ Jiangsu Product Quality Testing & Inspection Institute, 5 Guanghua Street, Nanjing , Jiangsu Province 210007, P.R. China

^3^ Jiangsu Key Laboratory for Food Quality and Safety/ Institute of Food Safety and Nutrition, Jiangsu Academy of Agricultural Sciences Nanjing 210014, P.R. China

^4^ Dongying Natural Resources and Planning Bureau, 95 Fuqian Street, Dongying, Shandong Province 257000, P.R. China.

Co-author: Song Yang, Yujun Xing

* Corresponding author: Ru Chen; chenrugss@163.com.

**Supplementary Materials**

Figure S1 The location of different ecological planting regions in China; Table S1 Different combinations of purifiers used in the purification; Table S2 Mass spectrometric parameters of 11 pesticides.


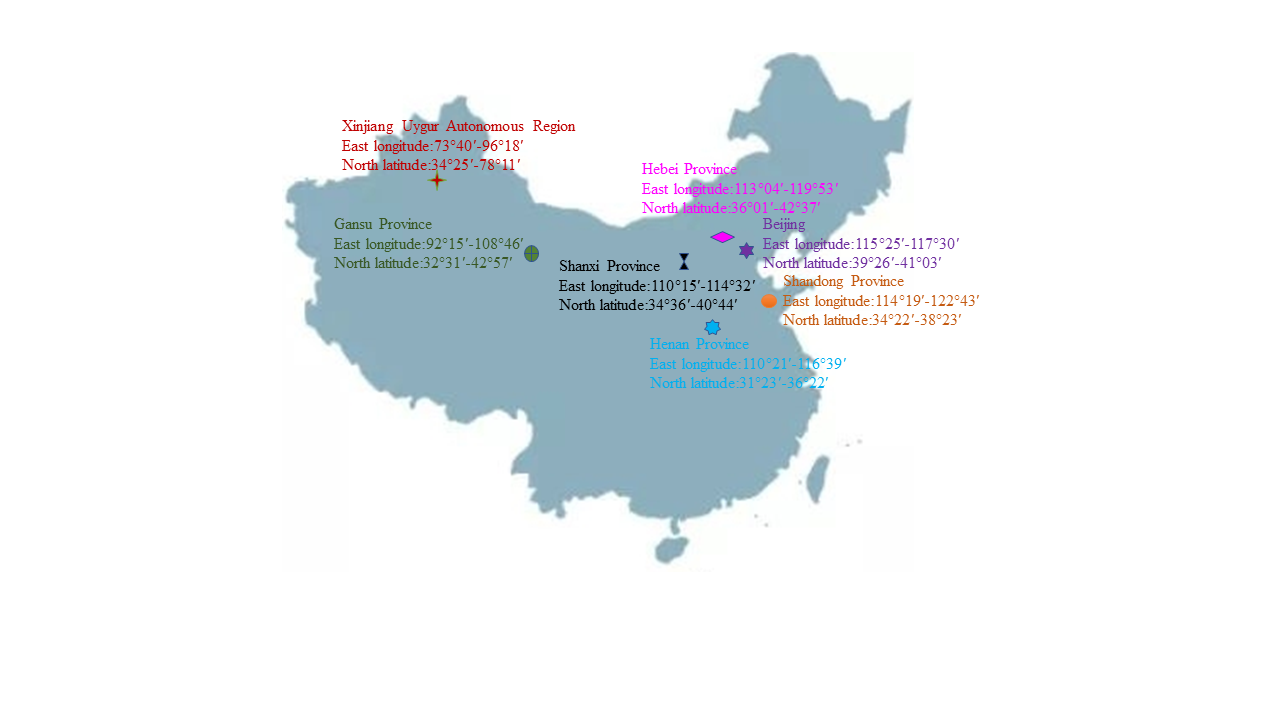


**Figure S1** The location of 7 provinces or cities of China.

**Table S1** Different combinations of purifiers used in the purification

| Purifier combination | Purifier dosage/mg |
| --- | --- |
| PSA/C_18_/MWCNTs | 10/40/5 |
|  | 25/25/5 |
|  | 40/10/5 |
| PSA/Nano-ZrO_2_/MWCNTs | 10/30/5 |
|  | 20/20/5 |
|  | 30/10/5 |
| C_18_/Nano-ZrO_2_/MWCNTs | 10/30/5 |
|  | 20/20/5 |
|  | 30/10/5 |

**Table S2** Mass spectrometric parameters of 11 pesticides

| Compounds | Retention Time (min) | Precursor Ion (m/z) | Quantification Ion (m/z) | Confirmation Ion (m/z) | Cone Voltage (V) | Collision Energy* (eV) | Collision Energy (eV) |
| --- | --- | --- | --- | --- | --- | --- | --- |
| 1 abamectin (B1a) | 5.98 | 890.5 | 305.2 | 567.3 | 34 | 25 | 14 |
| 2 imidacloprid | 1.21 | 256.1 | 209.1 | 175.1 | 29 | 14 | 17 |
| 3 chlorpyrifos | 3.58 | 351.9 | 97.0 | 199.9 | 10 | 35 | 20 |
| 4 *β*-cypermethrin | 4.98 | 433.1 | 191.1 | 127.1 | 28 | 15 | 29 |
| 5 phoxim | 4.56 | 299.0 | 77.1 | 129.1 | 30 | 26 | 10 |
| 6 procymidone | 4.71 | 284.1 | 256.1 | 67.1 | 42 | 28 | 17 |
| 7 acetamiprid | 1.07 | 223.1 | 126.1 | 56.1 | 30 | 22 | 15 |
| 8 deltamethrin | 5.04 | 523.0 | 181.0 | 280.9 | 45 | 49 | 16 |
| 9 fenpropathrin | 4.87 | 367.1 | 97.2 | 125.2 | 21 | 33 | 16 |
| 10 bifenthrin | 5.76 | 440.1 | 181.1 | 166.2 | 36 | 13 | 38 |
| 11 diflubenzuron | 4.23 | 309.1 | 289.1 | 156.2 | 21 | 8 | 10 |

Note: “*” is the collision energy of Quantification Ion.
